# Prompt Action by Voluntary Hospitals Essential

**Published:** 1917-12-01

**Authors:** 


### PROMPT ACTION BY VOLUNTARY HOSPITALS ESSENTIAL.

Much surprise was caused in the House of Commons on Friday, the 23rd ult., when the National Insurance Act Amendment Bill was down for second reading. That surprise was due to the fact that two clauses of this Bill having an important and direct bearing upon the interests of our voluntary hospitals, during a fairly exhaustive discussion, were not even referred to. Indeed, the second reading went through not only without remonstrance, but without even an inquiry for information, and a demand for redress.

The Bill in question appears to have entirely escaped the notice of hospital managers throughout the country, with one notable exception. This is unfortunate, but no time must be lost in finding some one or more members of the House of Commons to represent the interests of the voluntary hospitals, and to deal effectively with Clause 20, which is an important modification of the original Act affecting members of Approved Societies having no dependants, who are under treatment in hospitals. The old clause dealing with these points has been a source of very great uncertainty and contention. Some Approved Societies have administered the benefit in favour of hospitals, others, and amongst them the largest Society, have not done so. Under the new Clause 20 (2b) the sum which would otherwise be payable on account of any benefit to an insured contributor when an inmate of a hospital, if that person has no dependants, and with his authority, may in some eventualities be applied, *at the discretion of the Society or Committee administering the benefit*, in whole or in part to the institution of which he is an inmate. Section 12, sub-section (c) of the original Act provided that where a member of an Approved Society, being an inmate of a hospital, etc., supported by voluntary subscriptions, has no dependants, the benefit shall, if an agreement has been made between the Society and the Committee of the hospital, be paid in whole or in part under such agreement towards the maintenance of such persons in the hospital. Under Clause 20, section 2, sub-section (b) of the Amending Bill, the sickness benefit *may be applied at the discretion of the Society or the Committee in whole or in part* to the insured contributor's maintenance in an institution. We feel quite certain that the smooth working of the National Insurance Act will never be brought about whilst permissive provision on the part of Approved Societies forms part of the Act.

It is clear to us that hospitals and other institutions should give this important matter their immediate consideration with a view to prompt Parliamentary action. The Amending Bill should certainly have most careful weighing by hospital committees, and the British Hospitals Association might usefully co-operate with the object of securing the deletion of the words in Clause 20, and the equivalent words in sub-section (b), *i.e.*, "may be applied at the discretion of the Society or Committee administering the benefits towards defraying," etc., and the insertion in their place of "shall be applied towards defraying," thus making the provisions of the Act in their relations to hospitals and institutions not permissive but obligatory on the part of Approved Societies. There are other clauses affecting charitable homes which deserve the consideration of those immediately concerned.

We have often pointed out that the National Insurance Act has been saved from absolute failure by the voluntary hospitals. They must in justice, with all possible dispatch, be saved from the impossible position created by the Amending Bill, which places the financial recognition of their work at the discretion of Approved Societies.

